# On-scene selective brain cooling in ventricular fibrillation cardiac arrest: pilot results from the PRINCESS2 randomised trial

**DOI:** 10.1186/s13054-026-05851-y

**Published:** 2026-02-12

**Authors:** Emelie Dillenbeck, Thomas Berthelsen, Ervigio Corral Torres, Esteban López-de-Sa, Sandra O Rosillo Rodríguez, Miguel Sanchez-García, Juan Carlos Martín Benítez, Hans-Jörg Busch, Peter Radsel, Giuseppe Ristagno, Graham Nichol, Michael Holzer, Anthony Moreau, Sune Forsberg, Denise Bäckström, Akil Awad, Fabio Silvio Taccone, Anna Falk, Jacob Hollenberg, Martin Jonsson, Andreas Liliequist, Juliane Jurga, Mantas Okas, Leif Svensson, Per Nordberg

**Affiliations:** 1 Department of Clinical Science and Education, Center for Resuscitation Science, Karolinska Institutet, Södersjukhuset, Stockholm, Sweden; 2 Department of Physiology and Pharmacology , Karolinska Institutet , Stockholm, Sweden; 3 Perioperative Medicine and Intensive Care Function , Karolinska University Hospital , Stockholm, Sweden; 4 SAMUR Protección Civil , Madrid, Spain; 5 Cardiovascular Research Division , CARDIO-ELA , Madrid, Spain; 6 Department of Cardiology, Acute Cardiac Care Unit, Hospital Universitario La Paz, IdiPAZ, Madrid, Spain; 7 Critical Care Department , Hospital Clínico San Carlos , Madrid, Spain; 8 Department of Emergency Medicine, Faculty of Medicine, University Hospital of Freiburg,University of Freiburg, Freiburg, Germany; 9 Center for Intensive Internal Medicine , University Medical Center , Ljubljana, Slovenia; 10 Department of Anesthesiology, Intensive Care and Emergency, Fondazione IRCCS Ca’ Granda Ospedale Maggiore Policlinico, University of Milan, Milan, Italy; 11 University of Washington-Harborview Center for Prehospital Emergency Care , University of Washington , WA Seattle, USA; 12 Department of Emergency Medicine, Medical University of Vienna, Vienna, Austria; 13 Department of Intensive Care, Hôpital Universitaire de Bruxelles (HUB), Université libre de Bruxelles (ULB), Brussels, Belgium; 14 Department of Biomedical and Clinical Sciences , Linköping University , Linköping, Sweden; 15 Department of Medicine Solna, Karolinska Institutet, Stockholm, Sweden; 16 Department of Clinical Science, Intervention and Technology (CLINTEC), Karolinska Institutet, Stockholm, Sweden

**Keywords:** Out-of-hospital cardiac arrest, Ventricular fibrillation, Prehospital cooling, Therapeutic hypothermia, Neurological recovery

## Abstract

**Background:**

Although ischemia-reperfusion brain injury represents a major clinical problem after cardiac arrest, no neuroprotective treatment currently exists. The PRINCESS2 trial is designed to confirm previous findings, indicating that on-scene selective brain cooling using a portable trans-nasal cooling method improves complete neurological recovery after out-of-hospital cardiac arrest (OHCA) with initial shockable rhythms (e.g. ventricular fibrillation). This prespecified pilot phase aimed to assess protocol adherence and safety aspects for this early cooling strategy.

**Methods:**

The prespecified pilot phase includes the first 100 patients in the main PRINCESS2 trial, an ongoing European multicentre trial enrolling 1,022 OHCA patients with initial shockable rhythm. Patients are randomised to intervention; trans-nasal cooling initiated at the scene of arrest followed by systemic hypothermia (33 ± 0.5 °C for 24 h) in the intensive care unit, or control; normothermia with fever control. Neuroprognostication and criteria for withdrawal of life-sustaining treatment are protocolised. Pilot outcomes were adherence to treatment allocation and protocol in the prehospital and in-hospital stages, and safety aspects, including 72-hour survival, prehospital re-arrest, device-related adverse events, and adverse events within 7 days such as bleeding, sepsis, arrhythmias with hemodynamic compromise, or need for circulatory support.

**Results:**

In total, 100 patients were randomised (median age 64 years, 91% male) to intervention (*n* = 50) or control (*n* = 50). All intervention patients received allocated treatment. Four had minor trans-nasal cooling interruptions, and two had systemic cooling interruptions. Among controls, 48/50 received allocated treatment, while two crossed over to cooling. One control was lost to follow-up. Overall, adherence to allocation and treatment protocol was 92%. Survival to 72 h was similar (intervention: 32/50 [64%], control: 31/49 [63%]) as well as prehospital re-arrest rates (11/49 [22%] vs. 9/40 [23%]). No device-related serious adverse events occurred. Adverse event rates within 7 days were similar.

**Conclusion:**

In this pilot phase of the PRINCESS2 trial, on-scene trans-nasal cooling in OHCA patients with shockable rhythms was performed with high protocol adherence without safety concerns. The main trial will continue as planned.

**Trial registration:**

ClinicalTrials.gov: NCT06025123, registered Feb 1, 2023.

**Graphical abstract:**

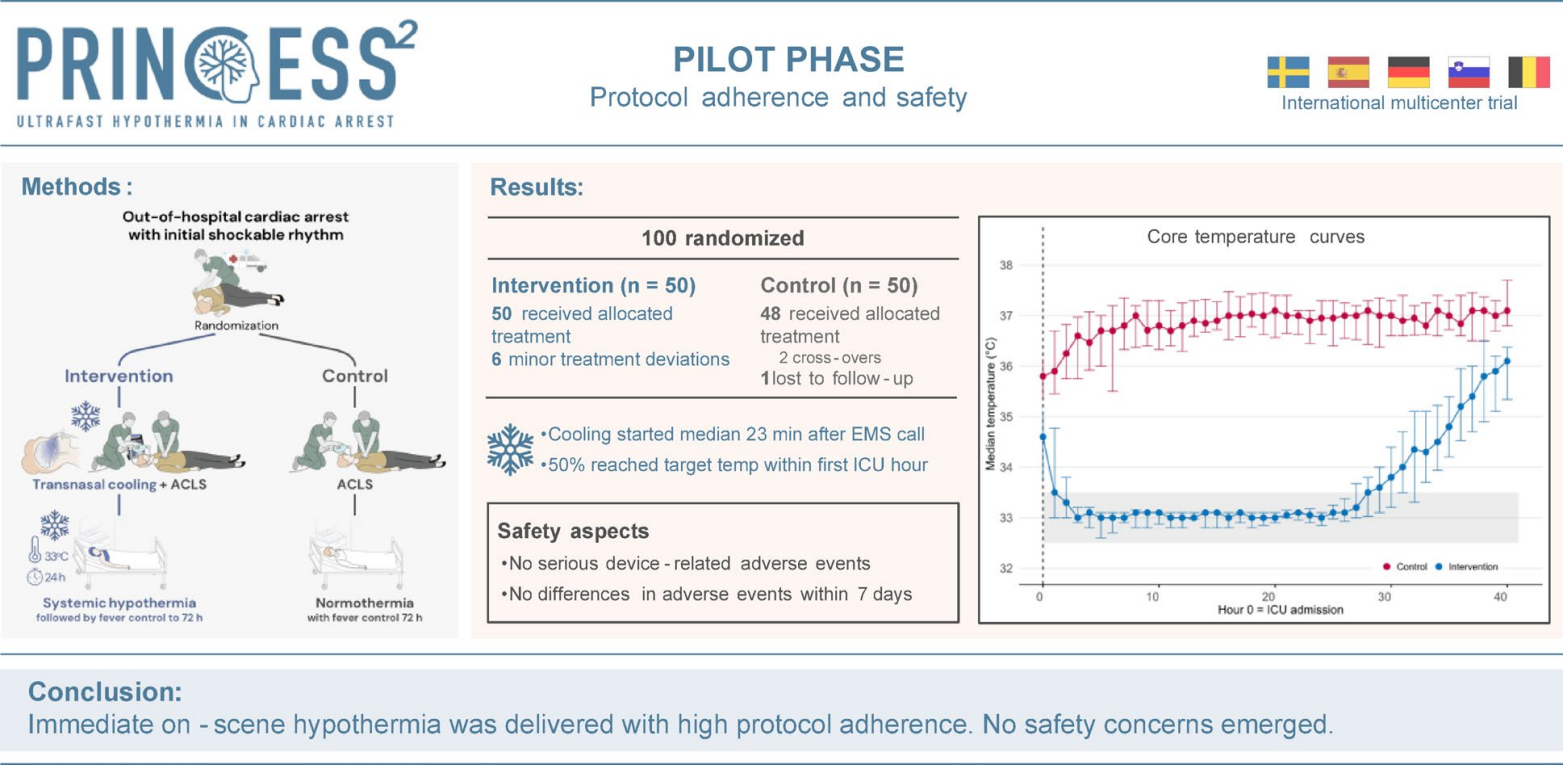

**Supplementary Information:**

The online version contains supplementary material available at 10.1186/s13054-026-05851-y.

## Background

Out-of-hospital cardiac arrest (OHCA) affects more than 300,000 patients annually in Europe, with an overall mortality of nearly 90% [1, 2]. Among resuscitated patients, almost two-thirds die in the hospital from ischemia-reperfusion brain injuries [3, 4], and no evidence-based neuroprotective treatment has been established. Therapeutic hypothermia may mitigate these injuries and improve neurological outcome after cardiac arrest [5], and experimental data suggests that the earlier hypothermia is initiated, the greater is the neuroprotective benefit [6–10].

However, clinical studies have yielded conflicting results. Two smaller randomised trials published in 2002 showed improved neurological outcomes with therapeutic hypothermia in a selected OHCA population with initial shockable rhythm [11, 12]. Similar benefits were found more recently in cardiac arrest patients with non-shockable rhythms [13]. In contrast, two large randomised trials studying a more heterogeneous OHCA population showed neutral results [14, 15]. A major limitation of all these clinical trials is the delayed initiation of cooling, typically starting after arrival to the intensive care unit (ICU), several hours post-arrest, potentially missing the critical therapeutic window for neuroprotection. Early cooling strategies initiated either during cardiac arrest or within minutes of return of spontaneous circulation (ROSC)– remain inadequately studied and have often been confounded by the negative effect of using large -volume cold intravenous fluids [16, 17]. The conflicting findings of therapeutic hypothermia have created uncertainty within the international scientific and clinical community, leading to significant variations in guidelines and clinical practices across hospitals and countries. The impact of immediate hypothermia initiated at the scene of collapse– is identified as a critical knowledge gap by international guideline organizations [18–20].

Trans-nasal evaporative cooling is a portable method developed to primarily cool the brain during resuscitation [21, 22]. The previous prehospital PRINCE and PRINCESS trials demonstrated that intra-arrest trans-nasal cooling is safe, feasible and reduces time to target temperature [21, 22]. Moreover, subgroup analyses suggested improved survival with complete neurological recovery in patients with shockable rhythms (e.g. ventricular fibrillation, pulseless ventricular tachycardia). Based on these findings, the ongoing PRINCESS2 trial aims to investigate whether on-scene selective brain cooling using trans-nasal cooling in OHCA patients with initial shockable rhythms, followed by systemic hypothermia in the ICU, significantly increases survival with complete neurological recovery compared to standard care, i.e. normothermia [23].

The PRINCESS2 trial design has some distinct differences from the preceding PRINCESS trial. The PRINCESS2 trial is only enrolling OHCA patients with initial shockable rhythm. In contrast to PRINCESS, it also includes patients who achieve ROSC on scene, thereby enhancing generalisability. It is also the first trial of trans-nasal cooling to include OHCA patients treated with extracorporeal cardiopulmonary resuscitation (ECPR), a subgroup in which both feasibility and protocol adherence remain unexplored. In addition, PRINCESS2 introduces a more comprehensive protocol for post resuscitation care, including a structured and blinded neurological prognostication prior to any decision on withdrawal of life-sustaining treatment.

Considering these modifications, we conducted this prespecified pilot phase including the 100 first patients from the main PRINCESS2 trial, with the primary aim to assess protocol adherence and safety aspects. Secondary aims were to evaluate protocol adherence and safety aspects in the ECPR subgroup, and to examine whether key aspects of randomisation and inclusion differed between centres.

## Methods

### Study design

The PRINCESS2 trial is an investigator-initiated, academic, international, multicentre, randomised (1:1) study enrolling 1022 patients with OHCA with initial shockable rhythm, investigating whether therapeutic hypothermia initiated at the scene of arrest using trans-nasal cooling improves survival with complete neurological recovery compared with normothermia with fever control. The study design has been described in detail previously [23]. This prespecified pilot phase evaluates protocol adherence and safety as part of the main trial, and all participants will be included in the main PRINCESS2 trial. The primary and secondary outcomes of the main trial are not analysed here.

The trial was approved by the ethics committees of all the participating sites and is registered at ClincalTrials.gov (NCT06025123). Due to the emergency nature of the intervention, enrolment occurred without prior informed consent. Written informed consent was obtained from all participants who regained mental capacity. In accordance with local ethics legislations, sites either used deferred consent for those who did not regain mental capacity, or obtained written consent from a legally authorised representative/next of kin [23].

### Settings

This prespecified pilot phase was conducted by multiple EMS systems and ICUs across five European countries (Sweden, Germany, Spain, Slovenia and Belgium). Participating hospitals provide 24/7 percutaneous coronary intervention (PCI) capability. A detailed list of centres is provided in Additional file 1. Inclusion began in Stockholm, Sweden, in April 2024, with other sites onboarding December 2024 to April 2025. The pilot phase concluded in July 2025 after the inclusion of 100 participants.

## Participants

The first 100 patients from the PRINCESS2 trial were included in the pilot phase. All participants from this pilot phase will be included in the final main trial analysis. Eligibility was identical to the main trial; patients were enrolled if all inclusion and no exclusion criteria were met.

### Inclusion criteria

1. Age ≥ 18 years with OHCA with initial shockable rhythm (i.e., ventricular fibrillation (VF), pulseless ventricular tachycardia (VT) or “shock advised” by an automated external defibrillator).
2. Unconsciousness (Glasgow Coma Scale ≤ 8).
3. Inclusion within 20 min from EMS arrival.

### Exclusion criteria

1. Age ≥ 80 years.
2. Obvious non-cardiac causes of cardiac arrest.
3. Obviously already hypothermic (e.g., found in the snow).
4. Obvious anatomic barrier to placing nasal catheters.
5. A written Do Not Attempt to Resuscitate order or a known terminal disease.
6. Known or clinically apparent pregnancy.

#### Randomisation and study intervention

Randomisation, and thus inclusion, was performed by EMS personnel at the scene of arrest, after the airway was secured (endotracheal intubation or supraglottic airway). Allocation was 1:1, using permuted blocks of varied size (4-8), stratified by trial site. The allocation sequence was generated by an investigator not involved in recruitment using the blockrand package in R, version 4.2.2 (R Foundation for Statistical Computing), and was concealed from other investigators. The sites received sets of sequentially numbered envelopes containing the randomisation assignments; an envelope was placed with the cooling device and replaced after each enrolment.

All patients received on-scene resuscitation and post-resuscitation care according to European guidelines [24, 25], with the exception for the study intervention.

In the intervention group, trans-nasal cooling was initiated at the scene of arrest via trans-nasal cooling (RhinoChill, BrainCool Inc, Lund, Sweden, previously described in detail [21, 22]) and continued until systemic cooling was established in the hospital (surface or intravascular). Core temperature was maintained at 33 ± 0.5 °C for 24 h, followed by controlled rewarming. Fever > 37.7 °C was actively treated to 72 h.

The control group received normothermia with active treatment of fever > 37.7 °C.

Participants in both groups were sedated for a minimum of 40 h. All admitted patients were treated actively for at least 72 h unless this was considered unethical (e.g. due to irreversible organ failure or advanced comorbidities). A standardised neurological prognostication for all participants still in the ICU at ≥ 72 h was performed by a physician blinded for group allocation. Follow-up at 90 days was performed by a blinded outcome-assessor.

### Study outcomes

The objectives of this pilot phase were to assess protocol adherence and safety. Protocol-adherence outcomes were defined as adherence to treatment allocation and to the treatment protocol in the prehospital and in-hospital phases (i.e. absence of interruption or discontinuation of the intervention). Acceptable overall protocol adherence, defined as adherence to both treatment allocation and the treatment protocol combined, was predefined as ≥ 90%. We also assessed adherence to sedation for 40 h, adherence to blinded prognostication at ≥ 72 h, number of patients randomised that were later found to match exclusion criteria, and the number of ICU-treated patients with withdrawal of life-sustaining therapy (WLST) before 72 h. For these latter exploratory outcomes, no formal adherence thresholds were prespecified; instead, they were considered exploratory and expected to fall within reasonable limits compared with previous studies. Safety outcomes were survival to 72 h, new prehospital cardiac arrest after inclusion and predefined adverse and serious adverse events within 7 days, including adverse device-related and unanticipated adverse device effects. Details on safety evaluation, AE/SAE definitions, and reporting procedures are provided in Additional file 1.

Secondary outcomes included the above mentioned outcomes for the ECPR subgroup, and in the overall population (both study groups combined), time to randomisation and the proportions of patients allocated to each group and enrolled intra-arrest versus post-ROSC at each centre. No outcomes from the main trial were analysed.

### Data collection

Data was reported in the different stages of the study; prehospital, in-hospital and 90-day follow-up. Data was recorded in electronic case report forms (eCRFs) (RED-Cap, Vanderbilt University, Nashville, TN, USA). Prehospital data was recorded by EMS personnel in close relation to the event. In-hospital data was recorded by physicians or research nurses. The results from the 90-day follow up were recorded by the outcome-assessor. Serious adverse events and device-related adverse events were reported in a special section of the eCRF which automatically alerts the principal investigator.

### Statistical analysis

The pilot sample size of 100 patients was chosen as reasonably appropriate to assess protocol adherence and safety aspects. Analyses followed the intention-to-treat (ITT) principle, including all randomised patients except the one patient lost to follow-up. No post-randomisation exclusions were made. Missing data were not imputed. Continuous variables are reported as medians with interquartile ranges (IQRs), and categorical variables are reported as counts and percentages. Due to the limited sample size in this pilot phase no comparative statistics were performed. Statistical analyses were made using R, version 4.2.2 (R Foundation for Statistical Computing).

## Results

**Fig. 1:**
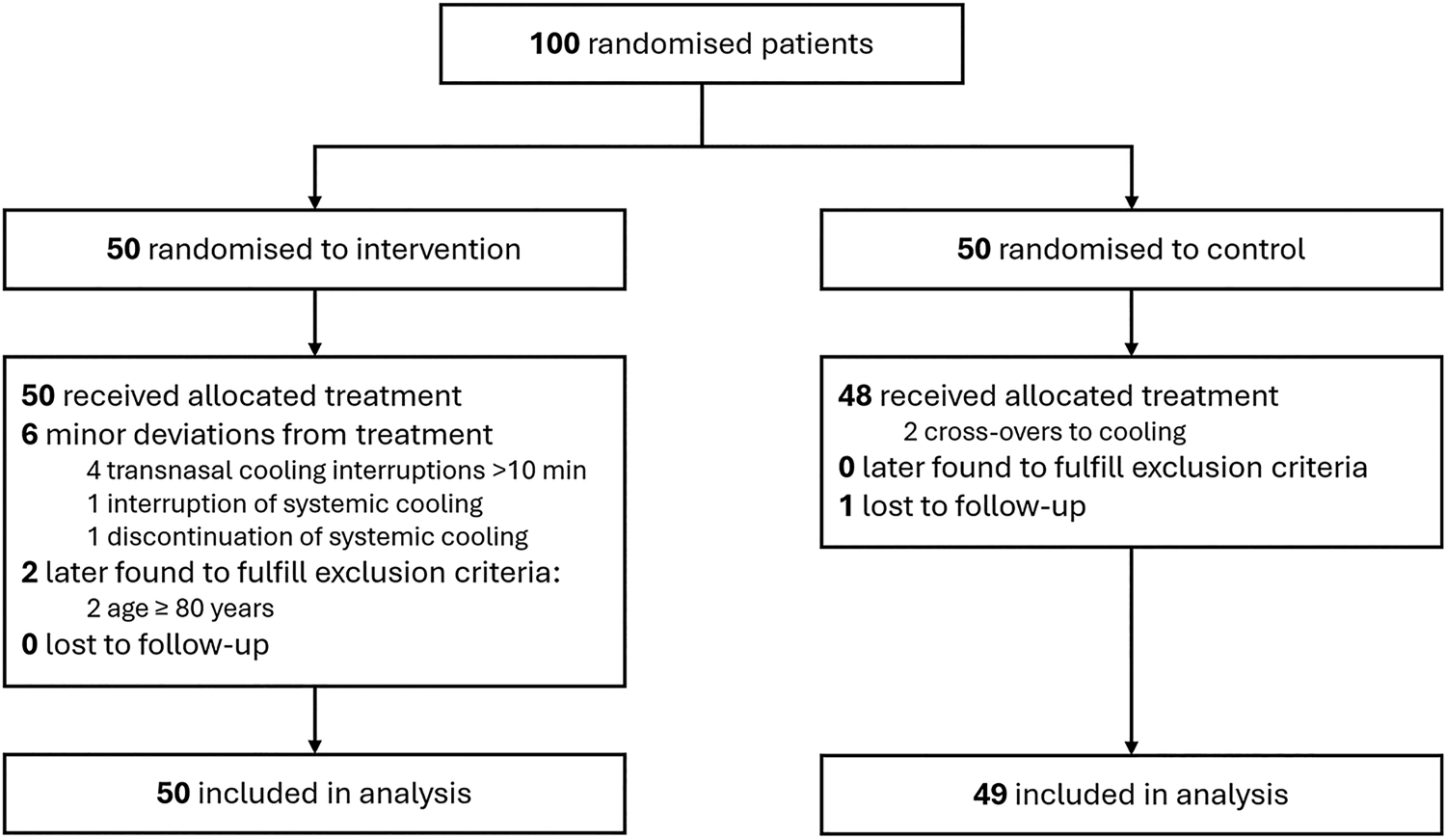
Flowchart of included patients

Of the 100 randomised patients, 50 were allocated to intervention and 50 to control. One control patient was transported to a non-study hospital and was lost to follow-up; therefore, 50 intervention patients and 49 control patients were included in the analysis (Fig. 1).

### Baseline characteristics, event times and post randomisation characteristics

Median age in the intervention group was 64 years (IQR 53, 71) and in the control group 64 years (IQR 52, 72). There were 46 (92%) men in the intervention group and 44 (90%) in the control group. Four intervention patients had an estimated pre-arrest modified Rankin Scale [26] (mRS) ≥ 2, compared with none in the control group. Median time from emergency call to secured airway and to randomisation was longer in the intervention group: 17 min (IQR 11, 23) and 19 min (IQR 16, 24) vs. 15 min (IQR 12, 20) and 16 min (IQR 13, 19), respectively. Other baseline characteristics and event times were similar between groups (Table 1).

**Table 1:** Baseline characteristics of the study population

	Treatment Groups	
	Intervention *n* = 50	Control *n* = 49
Patient Characteristics		
Age, median (IQR), y	64 (53, 71), (*n* = 50)	64 (52, 72), (*n* = 49)
Sex, n/total (%)		
Male	46/50 (92%)	44/49 (90%)
Female	4/50 (8.0%)	5/49 (10%)
Comorbidities, n/total (%)		
Ischemic heart disease	6/48 (13%)	10/48 (21%)
Previous Myocardial infarction	3/48 (6.3%)	5/48 (10%)
Heart failure	3/48 (6.3%)	3/48 (6.3%)
Atrial fibrillation	4/48 (8.3%)	6/48 (13%)
Hypertension	14/48 (29%)	18/48 (38%)
Diabetes Mellitus, type 1	1/48 (2.1%)	0/48 (0%)
Diabetes Mellitus, type 2	6/48 (13%)	7/48 (15%)
Chronic kidney disease	2/48 (4.2%)	2/48 (4.2%)
Cancer	2/48 (4.2%)	4/48 (8.3%)
Stroke	3/48 (6.3%)	3/48 (6.3%)
COPD	3/48 (6.3%)	3/48 (6.3%)
None	14/48 (29%)	13/48 (27%)
Estimated pre-arrest Clinical Frailty Scale, n/total (%)		
1	12/42 (29%)	7/39 (18%)
2	12/42 (29%)	13/39 (33%)
3	9/42 (21%)	14/39 (36%)
4	4/42 (9.5%)	4/39 (10%)
5	4/42 (9.5%)	0/39 (0%)
6	1/42 (2.4%)	1/39 (2.6%)
Estimated pre-arrest mRS, n/total (%)		
mRS 0–1	37/41 (90%)	36/36 (100%)
mRs ≥ 2	4/41 (9.8%)	0/36 (0%)
**Resuscitation characteristics**		
Location of cardiac arrest, n/total (%)		
Home	18/50 (36%)	13/47 (28%)
Public place	31/50 (62%)	32/47 (68%)
Other	1/50 (2.0%)	2/47 (4.3%)
Bystander CPR, n/total (%)	39/47 (83%)	38/47 (81%)
Airway, n/total (%)		
Laryngeal mask airway	9/50 (18%)	7/49 (14%)
Endotracheal tube	47/50 (94%)	40/49 (82%)
Laryngeal tube	2/50 (4.0%)	4/49 (8.2%)
Mechanical compression device, n/total (%)	27/49 (55%)	23/48 (48%)
Status at randomisation, n/total (%)		
Intra-arrest	33/48 (67%)	33/47 (70%)
Post ROSC	16/48 (33%)	14/47 (30%)
**Key timings**,** median (IQR)**,** min**		
Call^a^ to first EMS arrival	7 (4, 10), (*n* = 48)	6 (5, 8), (*n* = 46)
Call^a^ to second EMS arrival	12 (9, 15), (*n* = 41)	10 (7, 13), (*n* = 42)
Call^a^ to secured airway	17 (11, 23), (*n* = 47)	15 (12, 20), (*n* = 42)
Call^a^ to randomisation	19 (16, 24), (*n* = 48)	16 (13, 19), (*n* = 45)

COPD indicates Chronic obstructive pulmonary disease; CPR, cardiopulmonary resuscitation; EMS, emergency medical services. ^a^Emergency call

In the intervention group, trans-nasal cooling was initiated in median 23 min (IQR 21, 30) after emergency call. Temperatures differed between groups; for example, first registered core temperature was 34.7 °C (IQR 33.8, 35.5) in the intervention group vs. 35.8 °C (IQR 35.3, 36.5) in controls. Within in the first hour of ICU arrival, 14/28 (50%) of intervention patients had reached target temperature (33 ± 0.5 °C). Other characteristics and measures after hospital arrival were similar between the groups (Table 2).

**Table 2:** Post randomisation characteristics of the study population

	Treatment Groups	
Characteristic	Intervention *n* = 50	Control *n* = 49
**Prehospital characteristics**		
Call^a^ to start of cooling, median (IQR), min	23 (21, 30), (*n* = 47)	NA
Temperature at ROSC, median (IQR), ^o^C	35.7 (35.3, 36.4), (*n* = 22)	36.1 (35.3, 36.8), (*n* = 26)
Call^a^ to hospital arrival, median (IQR), min	49 (40, 68), (*n* = 46)	50 (39, 65), (*n* = 43)
**Inhospital characteristics**		
Tympanic temperature at hospital arrival, median (IQR), ^o^C	35.2 (34.0, 35.8), (*n* = 29)	35.8 (35.0, 36.5), (*n* = 28)
First registered core temperature^b^, median (IQR), ^o^C	34.7 (33.8, 35.5), (*n* = 31)	35.8 (35.3, 36.5), (*n* = 32)
Mean arterial pressure at hospital arrival, median (IQR), mmHg	87 (69, 99), (*n* = 32)	93 (73, 101), (*n* = 34)
Spontaneous breathing at hospital arrival, n/total (%)	7/40 (18%)	12/41 (29%)
First arterial blood gas^b^, median (IQR)		
pH	7.23 (7.11, 7.30), (*n* = 37)	7.23 (7.13, 7.31), (*n* = 41)
PO_2_, kPa	14 (8, 20), (*n* = 37)	11 (8, 17), (*n* = 41)
PCO_2_, kPa	6.2 (5.5, 7.4), (*n* = 37)	6.4 (5.4, 8.3), (*n* = 42)
Base excess, mmol/L	−10 (−14, −4), (*n* = 36)	−9 (−13, −3), (*n* = 39)
Lactate, mmol/L	6.7 (2.5, 10.0), (*n* = 37)	6.9 (4.3, 11.5), (*n* = 42)
Glucose, mmol/L	13.4 (10.7, 17.7), (*n* = 36)	13.9 (10.1, 16.0), (*n* = 39)
STEMI/new LBBB post ROSC, n/total (%)	17/37 (46%)	16/38 (42%)
Coronary angiography within the first 24 h, n/total (%)	25/40 (63%)	21/37 (57%)
PCI performed, n/total (%)	21/36 (58%)	20/37 (54%)
Call^a^ to ICU arrival, median (IQR), min	137 (88, 181), (*n* = 23)	131 (85, 192), (*n* = 27)
Core temperature ≤ 34 °C within first hour at ICU, n/total (%)	17/28 (61%)	0/19 (0%)
Core temperature ≤ 33.5 °C within first hour at ICU, n/total (%)	14/28 (50%)	0/19 (0%)
Fever treated with device hour 0–40^c^, n/total (%)	2/23 (8.7%)	7/31 (23%)
Fever treated with device hour 41–72^c^, n/total (%)	4/23 (17%)	6/31 (19%)

ECG indicates electrocardiogram; ICU, intensive care unit; LBBB, left bundle branch block; ROSC, return of spontaneous circulation; STEMI, ST-elevation myocardial infarction. ^a^Emergency call, ^b^Measured in emergency room or ICU, ^c^Fever defined as >37,7^o^C

A total of ten patients were treated with ECPR, 6 in the intervention group and 4 in the control group. ECPR-treated patients were younger than the overall cohort (median 48 years; IQR 46–52). Nine (90%) were male. Baseline characteristics and key timings for ECPR patients are presented in Additional file1, Table S1.

### Protocol adherence

**Fig. 2:**
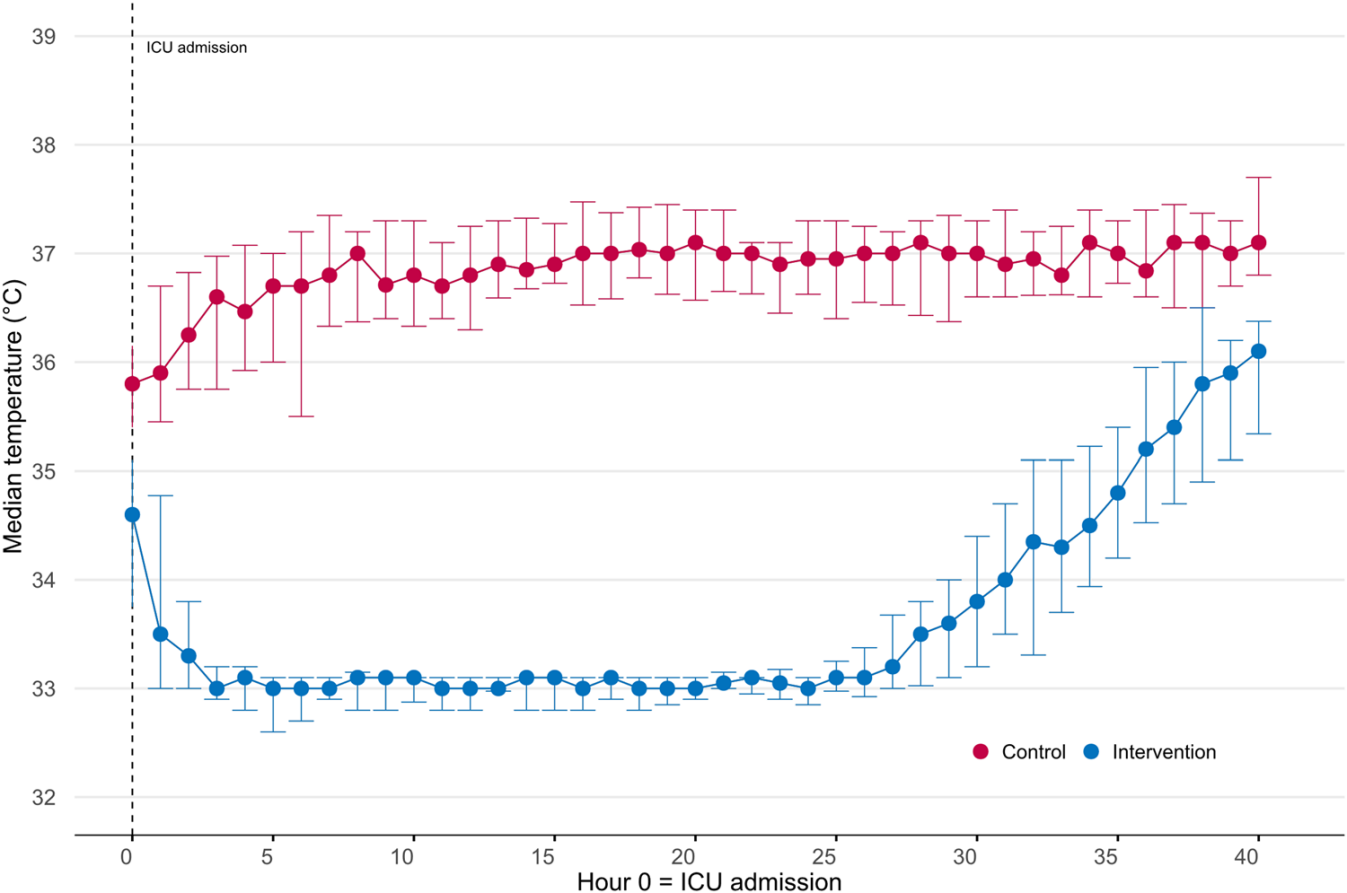
Body temperature during the first 40 h in the intensive care unit. The curves show the median (Q1-Q3) core body temperature. The median time from emergency call to intensive care unit (ICU) admission was 136 min

Adherence to allocation and treatment protocol combined was in total 92%. All patients in the intervention group received treatment according to allocation. In four patients, interruptions > 10 min of trans-nasal cooling occurred. In two cases, these interruptions were due to technical issues (difficulty securing the cooling device in the ambulance in one case and an unspecified technical issue in the other). In the remaining two cases, interruptions were due to nosebleed. There was one interruption in systemic cooling due to bleeding and systemic cooling was discontinued early in one patient because of circulatory instability due to distributive shock, both permitted by protocol, but reported as a treatment deviation for transparency. In the control group, 48 patients were treated according to allocation. There were two crossovers to hypothermia; one patient received cooling initiated at the scene of arrest and subsequent systemic hypothermia in the ICU, and one was systemically cooled in the ICU. Figure 2 shows median temperatures for the two study groups during the first 40 h in the ICU.

All patients in both groups who were unconscious at hospital arrival were sedated for 40 h according to protocol. WLST before 72 h in patients admitted to the ICU occurred in 3 patients in the intervention group and 7 patients in the control group. Neurological prognostication at ≥ 72 h were performed unblinded in one patient from each group. An overview of protocol deviations is found in Table 3. There were 2 patients in the intervention group later found to match exclusion criteria (age ≥ 80 years).

**Table 3:** Protocol deviations in the study population

	Treatment Groups	
	Intervention *n* = 50	Control *n* = 49
Interruption in trans-nasal cooling^a^		
Technical issues, n	2	NA
Nosebleed, n	2	NA
Interruption in systemic cooling		
Severe bleeding^b^, n	1	NA
Discontinuation of systemic cooling		
Circulatory instability, n^c^	1	NA
Cooling performed in control group		
Crossover, n	NA	2
Not sedated for a minimum of 40 h^d^	0	0
Neurologic prognostication at 72 h not performed according to protocol	1	1
WLST before 72 h^e^		
Multiorgan failure, n	2	4
Cardiac, n	0	2
Cerebral, n	1	1

WLST indicates withdrawal of life-sustaining treatment

^a^Interruption in trans-nasal cooling for > 10 minutes, ^b^Traumatic resuscitation injuries (hemothorax/tamponade/mammary artery injury); assessed as not intervention-related, attributed to mechanical chest compressions, ^c^Distributive shock, ^d^Excluding patients fully awake at hospital arrival and patients who died before 40 h, ^e^In patients admitted to the intensive care unit

###  Safety

Safety measures are shown in Table 4. At 72 h, 32 intervention patients (64%) and 31 control patients (63%) were alive. There were 11/49 (22%) new prehospital cardiac arrests in the intervention group and 9/40 (23%) in the control group. In the intervention group, four cases of minor epistaxis and two of nasal whitening were reported. During the first 7 days, bradycardia requiring pacing occurred in two intervention patients and none in controls, whereas ventricular tachycardia occurred in four controls and none in the intervention group. There were no other between-group differences in serious adverse events within 7 days, and no serious or unanticipated adverse device effects were reported.

**Table 4:** Safety measures in the study population

	Treatment Groups	
	Intervention *n* = 50	Control *n* = 49
Alive at 72 h from cardiac arrest, n/total (%)	32/50 (64%)	31/49 (63%)
New prehospital cardiac arrest	11/49 (22%)	9/40 (23%)
**Serious adverse events within 7 days**,** n/total (%)**		
Moderate bleeding^a^	3/35 (8.6%)	1/37 (2.7%)
Severe bleeding^a^	1/34 (2.9%)	2/35 (5.7%)
Sepsis and septic shock^b^	1/36 (2.8%)	1/37 (2.7%)
Cerebrovascular lesion^c^	3/36 (8.3%)	3/37 (8.1%)
Arrythmias with hemodynamic compromise		
Bradycardia with need for pacing	2/35 (5.7%)	0/36 (0%)
Ventricular tachycardia	0/35 (0%)	4/36 (11%)
Ventricular fibrillation	5/35 (14%)	5/36 (14%)
Need for circulatory support		
Inotropic support (no mechanical support)	7/35 (20%)	7/37 (19%)
Intra-aortic balloon pump	3/37 (8.1%)	1/38 (2.6%)
Impella	0/36 (0%)	1/38 (2.6%)
ECMO including ECPR	6/46 (13%)	7/46 (15%)
ECPR	6/43 (14%)	4/45 (8.9%)
**Reported adverse device effects**,** n**		
Minor nosebleed	4	0
Whitening of the nose	2	0
**Reported serious adverse device effects**,** n**	0	0
**Reported unanticipated adverse device effects**,** n**	0	0

ECMO indicates extracorporeal membrane oxygenation; ECPR, Extracorporeal cardiopulmonary resuscitation^a^According to the GUSTO criteria^b^According to the 3rd international consensus definitions for sepsis and septic shock. ^c^Caused by bleeding or infarction

### Secondary outcomes

Protocol adherence and safety aspects for the ECPR population are presented in Additional file 1, Table S2. WLST before 72 h in patients admitted to the ICU occurred in 1 patient in the intervention group and 3 patients in the control group. At 72 h, 2 intervention patients and 1 control patient were alive. Serious adverse events were similar between groups.

Key characteristics per centre are shown in Additional file 1, Table S3. In the centres recruiting > 5 patients, no imbalance in allocation, time to randomisation or status at randomisation was seen.

## Discussion

The ongoing PRINCESS2 trial is designed and powered to detect a significant difference in survival with complete neurologic recovery in OHCA with initial shockable rhythm, comparing therapeutic hypothermia initiated at the scene of arrest with normothermia. This prespecified pilot phase including the first 100 patients from the main trial evaluated protocol adherence and safety and found that adherence to protocol was high and there were no detected safety issues.

### Protocol adherence

In the intervention group, all patients received intervention as assigned. This differs from the prior PRINCESS trial [22], where 15/343 patients (4,4%) randomised to intervention did not receive trans-nasal cooling, mainly due to technical issues. In our pilot, there were four interruptions of trans-nasal cooling > 10 min (two due to technical problems) and a further seven reports of technical issues that did not cause significant interruptions. Reported problems were difficulty securing the device in the ambulance, low driving-gas levels, and challenges inserting nasal catheters. These technical issues will be closely monitored and feedback will be provided to the manufacturer, with additional user training offered as needed.

In the control group, two crossovers to cooling occurred: one patient received systemic cooling in the ICU owing to a misunderstanding, and another received on-scene plus systemic cooling at the treating EMS physician’s discretion. The multicentre design—spanning centres where routine care is hypothermic temperature control and others where it is normothermia with fever control—is a strength, but it necessitates thorough site training and reinforcement as needed to minimise crossovers.

Overall, temperature monitoring indicates that the intervention was delivered to a high standard. Half of intervention patients had reached target temperature within the first ICU hour. Due to difficulties in measuring core temperatures before ICU arrival at many centres, the median time to target temperature cannot be ascertained. The median time to ICU arrival in the intervention group was 137 min. In major trials evaluating hypothermia after OHCA, target temperature was typically achieved 5–8 h after randomisation, with randomisation itself occurring several hours post-arrest [13–15]. Thus, many of our patients reached target temperature substantially earlier—often before the time point at which patients in those trials were even randomised—underscoring the temporal advantage of on-scene initiation.

Use of a cooling device for fever control was lower in our control group than in the TTM2 trial control group (23% vs. 46%) [15]. Possible explanations include fewer patients reaching the prespecified fever threshold, adequate control with non-device measures (antipyretics, exposure, cold packs), or patients being awake/extubated before 72 h. Over the first 40 h, only four control patients had ≥ 1 fever measurement > 37.7 °C without cooling device treatment, and only one of them had fever for > 2 h, suggesting fever was well controlled.

Ten ICU-admitted patients were not treated for the full 72 h. This early WLST proportion was anticipated in this population and in line with the TTM2 trial results [15]. The main reasons for WLST were multiorgan and cardiogenic shock.

The high level of protocol adherence observed in this pilot phase likely reflects the participation of experienced and highly engaged study centres and underscores the importance of involving committed sites with experienced teams and a shared ambition to conduct the trial to a high standard.

#### Safety

Serious adverse events within 7 days in this pilot phase were consistent with expectations for this population and did not differ between groups. Previous prehospital cooling trials using rapid infusion of cold fluids have reported higher rates of re-arrest in the field [16]. We did not observe this signal in this pilot phase, nor was it seen in the prior PRINCESS trial [22]. Unlike cold-fluid cooling, which adds intravascular volume and may reduce coronary perfusion pressure, trans-nasal cooling is volume-neutral and may therefore not affect the risk of re-arrest. New prehospital cardiac arrest occurred in 22% in the intervention group and 23% in controls, slightly higher than the PRINCESS trial (intervention 16,8%, control 13,5%) [22] but similar to proportions observed in the control group of another prehospital cooling trial [16]. However, data review suggested that this variable may have been misunderstood and over-reported in some cases. Additional clarification will therefore be implemented in the prehospital eCRF. During the first 7 days of hospitalization, VT and VF occurred in 5 patients in the intervention group and 9 in the control group.

Minor nosebleed and whitening of the nose were reported at roughly half the frequency seen in PRINCESS [22]. Whether this reflects under-reporting or a true difference is unclear, and the eCRF will be adapted to better monitor this. No serious device related effects were reported. There was no imbalance in time to hospital or ICU arrival, suggesting the intervention did not delay on-scene care or pre-ICU in-hospital procedures.

### Secondary outcomes

In the ECPR subgroup, adverse events were proportionally more frequent than in the overall cohort, which is not surprising given the clinical instability of these patients. No imbalances of safety outcomes were found between the groups, although the small sample warrants caution in interpretation. ECPR patients are highly anticoagulated, and hypothermia might also affect coagulation, however, no severe bleeding was reported in the intervention group, and several other studies report no increased risk of bleeding with therapeutic hypothermia in ECPR [27, 28]. The time from hospital arrival to ECMO start was similar between the groups, suggesting the intervention did not delay time to ECMO. Early temperature data and timestamps were missing in more than half of ECPR cases, limiting assessment of cooling effectiveness.

#### Limitations

This pilot phase has several limitations. First, due to the nature of the intervention, neither EMS nor hospital staff could be blinded to treatment allocation. However, neurologic prognostication at ≥ 72 h and assessment of neurologic recovery was performed by blinded assessors. Second, no formal screening log was maintained (given the emergency setting and operational constraints), and variation of the inclusion rate over time was seen for some sites. Also, some sites planned for the main trial were not represented in the pilot phase since they had not yet commenced enrolment. These factors introduce a risk of selection bias and may affect generalisability to the main trial. However, most participating sites did include patients in the pilot phase, and they are distributed across Europe with diverse EMS and hospital systems, which may mitigate this risk. Finally, the sample size may be too small to detect clinically important differences in safety outcomes.

## Conclusion

In this prespecified pilot phase of the PRINCESS2 trial, on-scene trans-nasal cooling in OHCA patients with shockable rhythms was performed with high protocol adherence. No safety concerns emerged. The main trial will therefore continue as planned with no major protocol adjustments; minor eCRF clarifications will be introduced.

Supplementary Information.

## Supplementary Information

Supplementary Material 1

## Data Availability

The dataset generated and analysed during the current study are not publicly available due to data protection requirements within national law.

## Abbreviations

ECPR: Extracorporeal cardiopulmonary resuscitation
ECMO: Extracorporeal membrane oxygenation
eCRF: electronic Case Report Form
EMS: Emergency medical system
ICU: Intensive care unit
IQR: Interquartile range
mRS: modified Rankin scale
OHCA: Out-of-hospital cardiac arrest
VF: Ventricular fibrillation
VT: Ventricular tachycardia
WLST: Withdrawal of life-sustaining therapy

## References

[1] Gräsner JT, Wnent J, Herlitz J, Perkins GD, Lefering R, Tjelmeland I, et al. Survival after out-of-hospital cardiac arrest in Europe - Results of the EuReCa TWO study. Resuscitation. 2020;148:218–26. 32027980 10.1016/j.resuscitation.2019.12.042

[2] Empana JP, Lerner I, Valentin E, Folke F, Böttiger B, Gislason G et al (2022) Incidence of sudden cardiac death in the European Union. J Am Coll Cardiol 79(18):1818–27 35512862 10.1016/j.jacc.2022.02.041

[3] Lemiale V, Dumas F, Mongardon N, Giovanetti O, Charpentier J, Chiche JD et al (2013) Intensive care unit mortality after cardiac arrest: the relative contribution of shock and brain injury in a large cohort. Intensive Care Med 39(11):1972–80 23942856 10.1007/s00134-013-3043-4

[4] Witten L, Gardner R, Holmberg MJ, Wiberg S, Moskowitz A, Mehta S et al (2019) Reasons for death in patients successfully resuscitated from out-of-hospital and in-hospital cardiac arrest. Resuscitation 136:93–9 30710595 10.1016/j.resuscitation.2019.01.031 PMC6476296

[5] Arrich J, Schütz N, Oppenauer J, Vendt J, Holzer M, Havel C, et al. Hypothermia for neuroprotection in adults after cardiac arrest. Cochrane Database Syst Rev. 2023;5(5):Cd004128. 37217440 10.1002/14651858.CD004128.pub5 PMC10202224

[6] Nozari A, Safar P, Stezoski SW, Wu X, Henchir J, Radovsky A et al (2004) Mild hypothermia during prolonged cardiopulmonary cerebral resuscitation increases conscious survival in dogs. Crit Care Med 32(10):2110–6 15483422 10.1097/01.ccm.0000142700.19377.ae

[7] Nozari A, Safar P, Stezoski SW, Wu X, Kostelnik S, Radovsky A et al (2006) Critical time window for intra-arrest cooling with cold saline flush in a dog model of cardiopulmonary resuscitation. Circulation 113(23):2690–6 16769925 10.1161/CIRCULATIONAHA.106.613349

[8] Kuboyama K, Safar P, Radovsky A, Tisherman SA, Stezoski SW, Alexander H. Delay in cooling negates the beneficial effect of mild resuscitative cerebral hypothermia after cardiac arrest in dogs: a prospective, randomized study. Crit Care Med. 1993;21(9):1348–58. 8370299 10.1097/00003246-199309000-00019

[9] Abella BS, Zhao D, Alvarado J, Hamann K, Vanden Hoek TL, Becker LB. Intra-arrest cooling improves outcomes in a murine cardiac arrest model. Circulation. 2004;109(22):2786–91. 15159295 10.1161/01.CIR.0000131940.19833.85

[10] Zhao D, Abella BS, Beiser DG, Alvarado JP, Wang H, Hamann KJ et al (2008) Intra-arrest cooling with delayed reperfusion yields higher survival than earlier normothermic resuscitation in a mouse model of cardiac arrest. Resuscitation 77(2):242–9 18096292 10.1016/j.resuscitation.2007.10.015 PMC2391241

[11] Hypothermia after Cardiac Arrest Study Group (2002) Mild therapeutic hypothermia to improve the neurologic outcome after cardiac arrest. N Engl J Med 346(8):549–56 11856793 10.1056/NEJMoa012689

[12] Bernard SA, Gray TW, Buist MD, Jones BM, Silvester W, Gutteridge G et al (2002) Treatment of comatose survivors of out-of-hospital cardiac arrest with induced hypothermia. N Engl J Med 346(8):557–63 11856794 10.1056/NEJMoa003289

[13] Lascarrou JB, Merdji H, Le Gouge A, Colin G, Grillet G, Girardie P et al (2019) Targeted temperature management for cardiac arrest with nonshockable rhythm. N Engl J Med 381(24):2327–37 31577396 10.1056/NEJMoa1906661

[14] Nielsen N, Wetterslev J, Cronberg T, Erlinge D, Gasche Y, Hassager C et al (2013) Targeted temperature management at 33°C versus 36°C after cardiac arrest. N Engl J Med 369(23):2197–206 24237006 10.1056/NEJMoa1310519

[15] Dankiewicz J, Cronberg T, Lilja G, Jakobsen JC, Levin H, Ullén S, et al. Hypothermia versus normothermia after Out-of-Hospital cardiac arrest. N Engl J Med. 2021;384(24):2283–94. 34133859 10.1056/NEJMoa2100591

[16] Kim F, Nichol G, Maynard C, Hallstrom A, Kudenchuk PJ, Rea T, et al. Effect of prehospital induction of mild hypothermia on survival and neurological status among adults with cardiac arrest: a randomized clinical trial. JAMA. 2014;311(1):45–52. 24240712 10.1001/jama.2013.282173 PMC13045629

[17] Bernard SA, Smith K, Finn J, Hein C, Grantham H, Bray JE, et al. Induction of therapeutic hypothermia during out-of-hospital cardiac arrest using a rapid infusion of cold saline: the RINSE trial (Rapid Infusion of Cold Normal Saline). Circulation. 2016;134(11):797–805. 27562972 10.1161/CIRCULATIONAHA.116.021989

[18] Greif R, Bray JE, Djärv T, Drennan IR, Liley HG, Ng KC, et al. Education, Implementation, and Teams; and First Aid Task Forces. Circulation. 2024;150(24):e580–687. 2024 International Consensus on Cardiopulmonary Resuscitation and Emergency Cardiovascular Care Science With Treatment Recommendations: Summary From the Basic Life Support; Advanced Life Support; Pediatric Life Support; Neonatal Life Support. 10.1161/CIR.0000000000001288 39540293

[19] Hirsch KG, Amorim E, Coppler PJ, Drennan IR, Elliott A, Gordon AJ, et al. Part 11: post-cardiac arrest care: 2025 American Heart Association guidelines for cardiopulmonary resuscitation and emergency cardiovascular care. Circulation. 2025;152(16_suppl_2):S673-s718. 41122894 10.1161/CIR.0000000000001375

[20] Nolan JP, Sandroni C, Cariou A, Cronberg T, D’Arrigo S, Haywood K, et al. European resuscitation council and European society of intensive care medicine guidelines 2025: post-resuscitation care. Intensive Care Med. 2025;51(12):2213-88 10.1016/j.resuscitation.2025.110809. 41123621 10.1007/s00134-025-08117-3

[21] Castrén M, Nordberg P, Svensson L, Taccone F, Vincent JL, Desruelles D et al (2010) Intra-arrest transnasal evaporative cooling: a randomized, prehospital, multicenter study (PRINCE: pre-ROSC IntraNasal Cooling Effectiveness). Circulation 122(7):729–36 20679548 10.1161/CIRCULATIONAHA.109.931691

[22] Nordberg P, Taccone FS, Truhlar A, Forsberg S, Hollenberg J, Jonsson M et al (2019) Effect of trans-nasal evaporative intra-arrest cooling on functional neurologic outcome in out-of-hospital cardiac arrest: the PRINCESS randomized clinical trial. JAMA 321(17):1677–85 31063573 10.1001/jama.2019.4149 PMC6506882

[23] Dillenbeck E, Hollenberg J, Holzer M, Busch HJ, Nichol G, Radsel P et al (2024) The design of the PRINCESS 2 trial: a randomized trial to study the impact of ultrafast hypothermia on complete neurologic recovery after out-of-hospital cardiac arrest with initial shockable rhythm. Am Heart J 271:97–108 38417773 10.1016/j.ahj.2024.02.020

[24] Soar J, Böttiger BW, Carli P, Couper K, Deakin CD, Djärv T et al (2021) European Resuscitation Council guidelines 2021: adult advanced life support. Resuscitation 161:115–51 33773825 10.1016/j.resuscitation.2021.02.010

[25] Nolan JP, Sandroni C, Böttiger BW, Cariou A, Cronberg T, Friberg H et al (2021) European Resuscitation Council and European Society of Intensive Care Medicine guidelines 2021: post-resuscitation care. Intensive Care Med 47(4):369–421 33765189 10.1007/s00134-021-06368-4 PMC7993077

[26] Rankin J. Cerebral vascular accidents in patients over the age of 60. part II. prognosis. Scott Med J. 1957;2(5):200–15. 13432835 10.1177/003693305700200504

[27] Mecklenburg A, Stamm J, Angriman F, Del Sorbo L, Fan E, Soeffker G et al (2021) Impact of therapeutic hypothermia on bleeding events in adult patients treated with extracorporeal life support peri-cardiac arrest. J Crit Care 62:12–8 33227591 10.1016/j.jcrc.2020.11.008

[28] Duan J, Ma Q, Zhu C, Shi Y, Duan B. eCPR combined with therapeutic hypothermia could improve survival and neurologic outcomes for patients with cardiac arrest: a meta-analysis. Front Cardiovasc Med. 2021;8:703567. 34485403 10.3389/fcvm.2021.703567 PMC8414549

