# Supplementary material for: On-scene selective brain cooling in ventricular fibrillation cardiac arrest: pilot results from the PRINCESS2 randomised trial
Source: Crit Care. 2026 Feb 12;30:93. doi: 10.1186/s13054-026-05851-y (PMC12930594; doi:10.1186/s13054-026-05851-y)
Supplement: Supplementary file 1 — Supplementary Material 1 [file 13054_2026_5851_MOESM1_ESM.pdf]

ADDITIONAL FILE 1

SUPPLEMENTARY APPENDIX

PRINCESS²

ULTRAFAST HYPOTHERMIA IN CARDIAC ARREST

PILOT PHASE

Contents

List of Participating Sites in Pilot Phase 3

Adverse Events Definitions, Assessments and Reporting 4

Supplementary Tables 7

Table S1. Baseline characteristics and key timings for the ECPR population 7

Table S2. Safety measures and protocol deviations for the ECPR population 8

Table S3. Key characteristics per centre 9

List of Participating Sites in Pilot Phase

Sweden

Stockholm

Ambulanssjukvården i Storstockholm AB (AISAB)

Karolinska Universitetssjukhuset

Södersjukhuset

Germany

Freiburg

Malteser Hilfsdienst Freiburg

German Red Cross Freiburg, DRK Kreisverband Freiburg

Universitätsklinik Freiburg

Kandel

DRK Rettungsdienst Südpfalz GmbH, Rettungswache Kandel

Asklepios Südpfalzklinik

Spain

Madrid

SAMUR - Protección Civil

Hospital Universitario La Paz

Hospital Clínico San Carlos

Slovenia

Ljubljana

Community Health Centre Ljubljana - General Emergency Medical Care

University Medical Centre Ljubljana

Belgium

Brussels

Europe Hospitals, St-Elisabeth

Adverse Events Definitions, Assessments and Reporting

Adverse Event Definitions

An Adverse Event (AE) is any untoward medical occurrence in a subject.

A Serious Adverse Event (SAE) is any adverse event that:

a) leads to death
b) leads to a serious deterioration in the health of the patient that:
    1. results in a life-threatening illness or injury
    2. results in a permanent impairment of a body structure or a body function
    3. requires in-patient hospitalization or prolongation of existing hospitalization
    4. results in medical or surgical intervention to prevent permanent impairment to a body structure or a body function

Adverse Device Effects and Serious Adverse Device Effects are those AEs and SAEs that occur as an untoward or unintended response to a medical device. These events include those which result from insufficiencies or inadequacies in the Instructions for Use or deployment of the device as well as user error.

An Unanticipated Adverse Device Effect (UADE) is defined as any serious adverse effect on health or safety or any life-threatening problem or death caused by – or associated with – the device, if that effect, problem or death was not previously identified in nature, severity, or degree of incidence in the investigational plan (including documents such as the protocol, Investigator's Brochure, informed consent form or other study-related documents), or any other unanticipated serious problem associated with the device that relates to the rights, safety or welfare of subjects.

A Technical Device Failure is defined as a failure of the device to perform its intended function when used in accordance with the Instructions for Use. Technical device failures will be recorded and evaluated for possible untoward effects on the subject. If a device failure results in an adverse experience in the subject, this adverse experience should be considered an adverse device effect and recorded on the Adverse Event pages of the CRF. Device failures that do not result in a clinically significant adverse effect on the patient will be noted on the CRF pages regarding device performance but will not be considered an adverse device effect.

Adverse Event Assessments

The relation of the event to the investigational device will be categorized by the Investigator as follows:

Not related – AE is due to the underlying disease state or concomitant medication or therapy, and was not caused by the investigational device.

Probably not related– AE had minimal or no temporal relationship to the use of the investigational device and/or a more likely alternative etiology exists.

Probably related – AE had a strong temporal relationship to the use of the investigational device and an alternative etiology is less likely compared to the potential relationship to the investigational device.

Definitely related – AE had a strong temporal relationship to the use of the investigational device and another etiology is highly unlikely.

For the purposes of reporting, an event will be considered associated with the use of the device if it is believed to be due either directly to the mechanical aspects of the device itself (e.g., nosebleed) or the ensuing device-related cooling.

Events believed to be due to study procedures other than the device/cooling (such as events believed to be side effects of the standard hypothermia maintenance) will be recorded but will not be categorized as device-related.

Subjects enrolled in the study will have a high morbidity and mortality rate associated with their cardiac arrest and the ensuing global ischemia. Therefore, careful attention shall be made to assessing the causality of any serious adverse events.

Adverse Events Reporting

All clinically significant AEs or those that appear to be related to the use of the RhinoChill (e.g., whitening of the nose) as well as those that could potentially harm the patient (e.g. barotrauma) will be recorded in a special section of the CRF from ROSC through the first 24 hours from inclusion. Abnormal laboratory values are expected in these patients, and these are not to be recorded as AEs. The date of occurrence, severity, duration, management, technical issues and relationship to cooling with the RhinoChill Device will be recorded.

Serious Adverse Event Reporting

All SAEs listed below are considered as common complications after cardiac arrest and do not need to be reported other than in the CRFs. These complications that occur within seven days after enrolment will be presented to the DSMB at the time of the interim analysis and presented in the main publication.

SAEs in both groups (thus not specifically device related) that should be reported in the CRF:

- New cardiac arrest after enrollment.
- Arrhythmias resulting hemodynamic compromise
- Bradycardia necessitating pacing
- Cerebrovascular lesion during ICU stay (bleeding or infarction)
- Sepsis and septic shock, according to the 3rd international consensus definitions for sepsis and septic shock
- Moderate or severe bleeding, according to the GUSTO criteria

Unanticipated Adverse Device Effect (UADE) and other unexpected SAE should be followed until resolution; this includes those patients that were terminated early or withdrawn. These must be reported to the study sponsor at The Karolinska Institutet (see contact information with email and phone to the principal investigator and study coordinator on the first page of the protocol) and at the same time to the company BrainCool AB as soon as possible, preferably within 24 hours of their occurrence as well as following their resolution. SAE:s such as those listed below will be reported in a separate electronic CRF in the database. When inserting data regarding adverse events in the database, the PI will automatically receive an email with information of the adverse event.

- Device related skin complications (blistering or skin necrosis)
- Device related bleeding requiring transfusion
- Barotrauma such as pneumocephalus
- Other, unexpected serious adverse events

The Principal Investigator, Per Nordberg and senior advisor professor Leif Svensson at the Department of Clinical Science and education, Karolinska Institutet, will review all SAE reports as soon as possible with regard to their causal relationship to use of the cooling method.

Reporting to the regulatory authorities will be performed per European vigilance requirements and other local requirements. This is a responsibility of the PI.

Supplementary Tables

Table S1. Baseline characteristics and key timings for the ECPR population

	Intervention<br>n = 6	Control<br>n = 4
**Baseline characteristics**		
Age, median (IQR), y	47 (41, 63), (n=6)	50 (47, 52), (n=4)
Sex, n/total (%)		
Male	6/6 (100%)	3/4 (75%)
Female	0/6 (0%)	1/4 (25%)
Location of cardiac arrest, n/total (%)		
Public place	2/6 (33%)	1/4 (25%)
Home	4/6 (67%)	2/4 (50%)
Other	0/6 (0%)	1/4 (25%)
Bystander CPR, n/total (%)	4/5 (80%)	3/4 (75%)
Status at randomization, n/total (%)		
Intra-arrest	6/6 (100%)	3/4 (75%)
Post ROSC	0/6 (0%)	1/4 (25%)
**Key timings**		
Call[a] to first EMS arrival	7.5 (6.0, 12.0), (n=6)	7.0 (6.0, 8.0), (n=4)
Call[a] to second EMS arrival	11.0 (7.5, 15.0), (n=4)	10.5 (9.0, 12.5), (n=4)
Call[a] to secured airway	17.5 (12.0, 19.0), (n=6)	16.5 (14.5, 19.0), (n=4)
Call[a] to randomisation	22.0 (16.0, 27.0), (n=6)	16.5 (13.0, 21.0), (n=4)
Call[a] to hospital arrival	47 (38, 55), (n=6)	45 (39, 55), (n=4)
Call[a] to start of cooling	29.0 (23.0, 29.0), (n=5)	NA
Call[a] to ICU arrival	162 (75, 275), (n=3)	170 (85, 254), (n=2)
Hospital arrival to start of ECMO	27 (19, 46), (n=5)	20 (18, 33), (n=3)

CPR indicates cardiopulmonary resuscitation; ECMO, extracorporeal membrane oxygenation; ECPR, extracorporeal cardiopulmonary resuscitation EMS, emergency medical services; ICU, intensive care unit
[a]Emergency call

Table S2. Safety measures and protocol deviations for the ECPR population

	Treatment Groups	
	**Intervention n = 6**	**Control n = 4**
Alive at 72 hours from cardiac arrest, n/total) (%)	2/6 (33%)	1/4 (25%)
**Serious adverse events within 7 days, n/total (%)**		
Moderate bleeding[a]	2/5 (40%)	1/3 (33%)
Severe bleeding[a]	0/5 (0%)	1/3 (33%)
Sepsis and septic shock[b]	1/5 (20%)	0/3 (0%)
Cerebrovascular lesion[c]	1/5 (20%)	1/3 (33%)
Arrythmias with hemodynamic compromise		
Bradycardia with need for pacing	0/4 (0%)	0/3 (0%)
Ventricular tachycardia	0/4 (0%)	0/3 (0%)
Ventricular fibrillation	2/4 (50%)	1/3 (33%)
**Reported adverse device effects, n**	0	0
**Reported serious adverse device effects, n**	0	0
**Reported unanticipated adverse device effects, n**	0	0
**Protocol deviations, n**		
Interruption in trans-nasal cooling[d]		
Technical issues	1	NA
Interruption in systemic cooling[e]	0	NA
WLST before 72 hours[f]		
Multiorgan failure	0	2
Cerebral	1	1

ECPR indicates extracorporeal cardiopulmonary resuscitation, WLST, withdrawal of life-sustaining treatment
[a]According to the GUSTO criteria
[b]According to the 3rd international consensus definitions for sepsis and septic shock
[c]Caused by bleeding or infarction
[d]Interruption in trans-nasal cooling for > 10 minutes
[e]Interruption in systemic cooling for any reason
[f]In patients admitted to the ICU. Allowed by protocol but reported as deviation for transparency

Table S3. Key characteristics per centre

	Centre						
	**Overall n = 99**	**Stockholm n = 46**	**Freiburg n = 5**	**Brussels n = 2**	**Ljubljana n = 9**	**Madrid n = 34**	**Kandel n = 3**
Call[a] to randomisation, median (IQR), min	17 (14, 22) (n= 93)	17 (14, 19) (n=42)	23 (17, 24) (n=5)	20 (16, 23) (n=2)	17 (14, 21) (n=9)	18 (16, 27) (n=32)	19 (13, 22) (n=3)
Randomised_to cooling, n/total (%)	50/99 (51%)	22/46 (48%)	1/5 (20%)	2/2 (100%)	6/9 (67%)	17/34 (50%)	2/3 (67%)
Status at randomisation, n/total (%)							
Intra-arrest	66/96 (69%)	33/44 (75%)	1/5 (20%)	0/2 (0%)	7/9 (78%)	23/33 (70%)	2/3 (67%)
Post ROSC	30/96 (31%)	11/44 (25%)	4/5 (80%)	2/2 (100%)	2/9 (22%)	10/33 (30%)	1/3 (33%)
Unknown	3	2	0	0	0	1	0

ROSC indicates return of spontaneous circulation
[a]Emergency call
